# Landslide assessment through integrated geoelectrical and seismic methods: A case study in Thungsong site, southern Thailand

**DOI:** 10.1016/j.heliyon.2024.e24660

**Published:** 2024-01-13

**Authors:** C. Sujitapan, J.M. Kendall, J.E. Chambers, S. Yordkayhun

**Affiliations:** a School of Sciences, Walailak University, Thaiburi, Thasala District, Nakhon Si Thammarat, 80161, Thailand; b University of Oxford, Department of Earth Sciences, Oxford, OX1 3AN, United Kingdom; c British Geological Survey, Keyworth, Nottingham, NG12 5GG, United Kingdom; d Geophysics Research Centre, Department of Physics, Faculty of Science, Prince of Songkla University, Hat Yai, Songkhla, 90112, Thailand

**Keywords:** Landslide, Electrical resistivity tomography (ERT), Seismic refraction tomography (SRT), 2D cross-plot of P-Wave velocity and resistivity values, Volume of sliding material

## Abstract

Many landslides can cause significant damage to infrastructure, property, and human life. To study landslide structure and processes, geophysical techniques are most productive when employed in combination with other survey and monitoring tools, such as intrusive sampling. Here, the integration of electrical resistivity tomography (ERT) and seismic refraction tomography (SRT) methods is used to assess landslides in Thungsong district, Nakhon Si Thammarat, the south of Thailand, where is a hilly and seasons of prolonged rainfall region. The 2D cross-plot analysis of P-wave velocity and resistivity values obtained by these two methods is introduced to identify potential landslide-prone zones in this region. The results of the 2D cross-plot model reveal detailed image of the subsurface conditions, highlighting areas of low P-wave velocity (lower than 600 m/s) and low resistivity (lower than 600 Ωm). These areas are indicative of weak zone and are potential to be sliding materials. Moreover, an intrusive sampling data from boreholes is also used for the calibration and validation geophysical data with geological data. This can improve the accuracy of landslide assessment and develop effective mitigation strategies to reduce the risk of landslides in this area. In addition of the 2D cross-plot, the volume of sliding material is also determined from the difference of the surface and slipping plane elevations. The volume calculation of sliding material is roughly 33447.76 m^3^. This approach provides a preliminary tool for landslide studies and monitoring landslides in this region, thus enabling an improved understanding of slope failure processes in this context, and the basis of a landslide mitigation strategy in the future.

## 1 Introduction

**Fig. 1:**
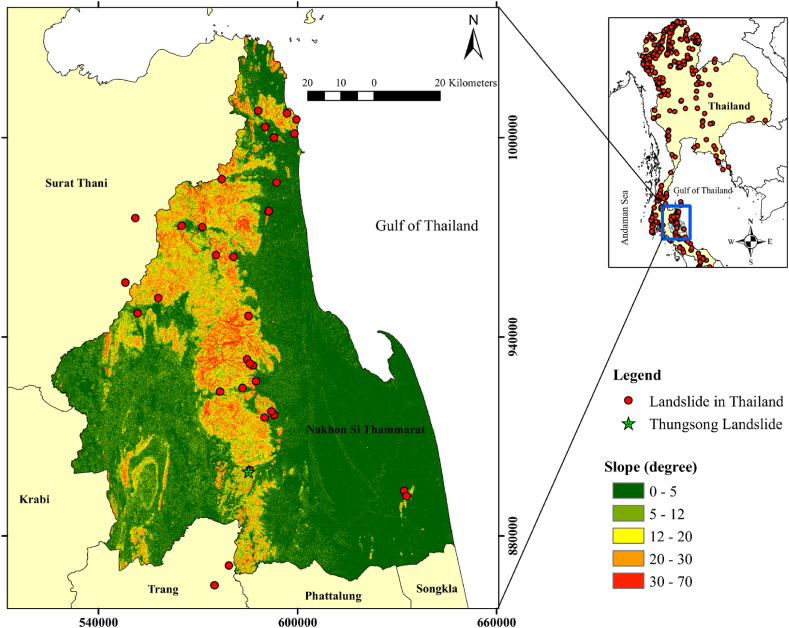
The slope angle map of Nakhon Si Thammarat province (blue rectangle) with landslide events (red circle). The location of Thungsong landslide represents in light green star. (For interpretation of the references to color in this figure legend, the reader is referred to the Web version of this article.)

Landslides are known as one of the most catastrophic types of natural hazards [1,2]. They can significantly damage to infrastructure and property, as well as pose a threat to human life [[3], [4], [5]]. Landslides are complex and diverse that exhibits various mechanism of mass movement downward on slopes under gravity force [6,7]. The landslide activations are widespread in many regions due to a growing population, increasing of land use, and the global climate changes related to rise in intense weather events [[8], [9], [10]]. Especially, extreme weather events with heavy rainfall that affects the movement of groundwater are very important factor in slope instability because fluctuations in moisture content directly affect pore pressure, leading to shear strength reduction in the subsurface [11,12]. Thailand is a country where often experiences the landslide hazards. The Department of Mineral Resource (DMR), Thailand has developed a database of landslide events in Thailand from 1988 to 2020 (Fig. 1). It reveals that major landslides mostly occurred in northern and southern parts of Thailand, due to mountainous terrain and hilly topography (slope angle more than 30°) (Fig. 1). Particularly in the southern part there were prolonged precipitation with mean annual rainfall intensity more than 2000 mm [13,14]. Furthermore, the other factors contributing to large landslides in southern Thailand are deforestation and mismanagement of land use due to a population growth. Therefore, there has been a rapidly increase in the landslide frequency from 1988 to the present [15]. The worst landslide events in this region emerged in November 1988 after extremely heavy rainfall and flooding in Kathun district, Nakhon Si Thammarat province. There were roughly 230 fatalities [16]. Moreover, in January 2017, there were more than five landslides in Thungsong area due to the torrential rainfall. The landslides destroyed many rubber plantations, which is an economic crop and obstructed the highway No. 41, which is the main road for transportation in the south of Thailand. They also damaged the walls of local houses in front of the hills [17].

To effectively mitigate the risk of landslides, it is important to understand the internal structures and mechanism of landslide contributing to their occurrences and to develop appropriate strategies for prevention and management. Geophysical methods, such as resistivity and seismic methods are advantageous for investigating landslides [18,19], because they can reveal comprehensive of large volume of subsurface in terms of the internal structure and physical characteristics. Furthermore, they are inexpensive and involve relatively fast field data acquisition compared to other methods [20,21]. However, the geophysical methods are most effective when they are combined with borehole and SPT-test data [7,14]. Here, P-wave seismic refraction tomography (SRT) and electrical resistivity tomography (ERT) methods are applied to study landslide. The P-wave SRT method is utilized to characterize subsurface structure, depth of shear zone, bedrock, and thickness of sliding mass in landslide area [7,22,23]. Unstable materials exhibit variation in physical properties of the subsurface, which can be seen as changes in P-wave velocities [24]. The ERT method is applied to investigate resistivity values of materials in the subsurface [25]. The resistivity value is sensitive to changes in lithology particularly moisture and clay content in the subsurface. Therefore, the ERT has been successful to delineate landslide structures in terms of the contrast between unstable and stable material, depth to bedrock, and composition of the subsurface [18,26]. In particular, the ERT exhibits notable effectiveness in assessing the distribution of groundwater and the moisture and clay content across various layers within the slope [[27], [28], [29], [30]]. Thus, the combination of P-wave SRT and ERT using a 2D cross-plot analysis approach can be used to be robust for evaluating landslides.

In landslide assessment, the 2D cross-plot analysis of P-wave velocity and resistivity values can improve subsurface resolution, facilitating the identification of slipping regions and the characterization of triggering factors contributing to slope fialure in this study [[31], [32], [33]]. The research area is Thungsong landslide (located in Nakhon Si Thammarat province, the south of Thailand (Fig. 1). According to the Cruden and Varnes (1996) [34], the landslides in the site are classified as shallow landslide with debris flow. The shallow landslides in this site occur as a consequence of the development of slip planes within the near surface. This is due to rainfall infiltration through the subsurface, unsaturated percolation, and rapid rise of the ground water table level [17]. Shallow failure emerges along a slip plane when the water content is saturated due to the accumulated rainfall leading to dissipated matric suctions and reduce of soil strength [35]. Consequently, the purpose of this study is to reveal the use of SRT and ERT with the 2D cross-plot model to investigate internal structure and mechanisms of landslide in a remote and challenging-to-reach location. The 2D Cross-Plot Analysis is aimed to identify sliding plane in landslide areas by analysing two key parameters: P-wave velocity and resistivity. Integration of these methods conjunction with borehole data is a pioneering effort in this geological setting of landslides, particularly in areas affected by monsoonal rainfall and characterized by mountainous terrain.

## 2 Geological settings and climatic conditions in research area

**Fig. 2:**
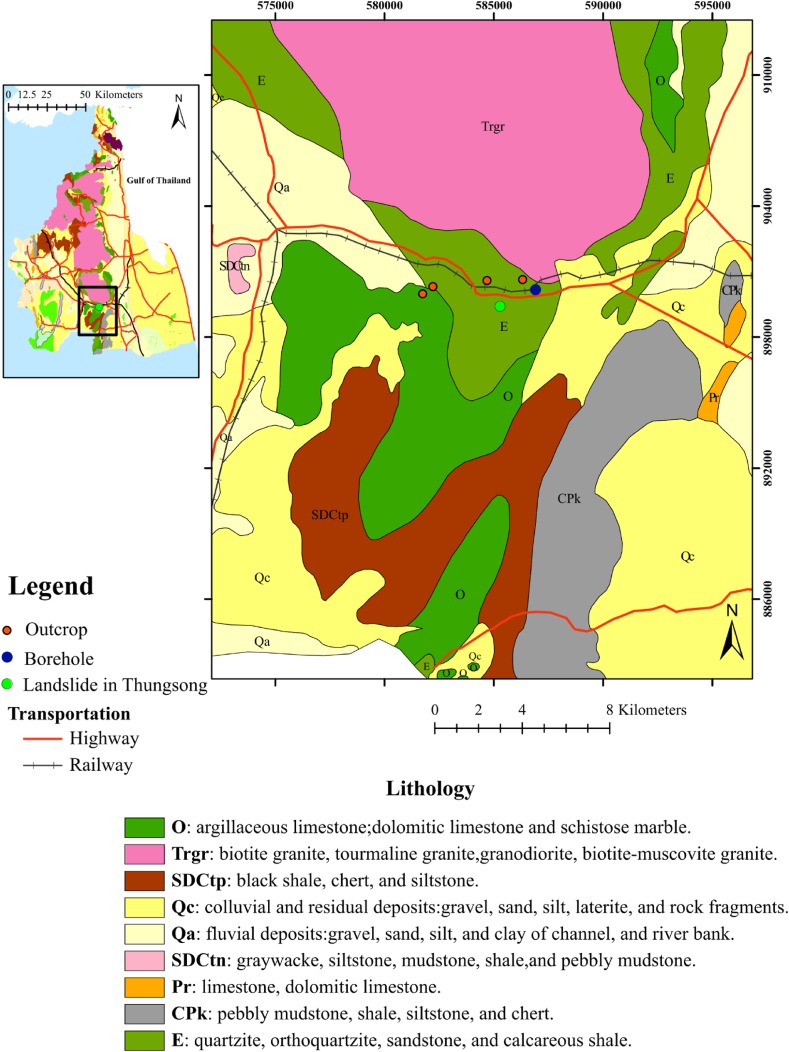
Geological map of Nakhon Si Thammmarat province (top left) and the study area (light green circle). (For interpretation of the references to color in this figure legend, the reader is referred to the Web version of this article.)

This landslide is located in Nakhon Si Thammarat province, southern Thailand (Fig. 1). This province lies on the east coast of the Thai Peninsula, known as Gulf of Thailand [36]. The landforms comprise two parallel mountain ranges, which are the Phuket and Nakhon Si Thammarat ranges in north–south direction [17,35]. Alluvial deposits, colluvium can be found in foothills alongside the ranges [36]. This landslide was occurred at the foothills of Nakhon Si Thammarat ranges, which is granitic rock [37] (pink area marked ‘Trgr’ in Fig. 2). The altitude of this area is between 150 and 180 m. The angles of slope in the study area are particularly steep in the range of 35–45° (Fig. 1). The bedrock geology in Nakhon Si Thammarat province (Fig. 2) consists of Cretaceous to Tertiary igneous rocks and Cambrian to Carboniferous sedimentary rocks. The landslide site is located in Cambrian sandstone bedrock, which is covered by colluvium and residual deposits in Quaternary age (dark yellow area marked ‘Qc’ in Fig. 2). Many landslides have emerged in this old sedimentary rock, primarily attributed to the accelerated weathering of sandstone and quartzite.

The succession of the bedrock in this area have been investigated by the outcrops (orange circles in Fig. 2). The Cambrian sedimentary rock Groups (light green area marked ‘E’ in Fig. 2) is classified in the part of Tarutao Group, which is characterized by thin- to medium-bedded layers of whitish-grey and greenish-grey quartzite, exhibiting well-sorted characteristics along with both cross and graded bedding in the sandstone layers [38]. This Group is overlain by the Thungsong Groups (dark green area marked ‘O’ in Fig. 2), which is an Ordovician limestone [38,39]. This Group is characterized by the presence of muddy limestone interspersed with dolomitic limestone layers. The top of the Thungsong Group is a combination of grey mudstone interspersed with very fine-grained siltstone and sandstone layers [38]. These two Groups are mantled by Quaternary colluvium, formed through weathering of the bedrock and accumulation at the base of a steep slope. Generally, the colluvium deposits vary in compositions associated with the type of bedrocks. Therefore, the colluvium deposits in this site are silt, clay, sand, gravel, and lateritic soil. Furthermore, there is a borehole near the study area (blue circle in Fig. 2), drilled by DGR (Department of Groundwater Resources). The borehole descriptions in Table 1 can contribute to a more comprehensive understanding of the lithology in this study area. At 0–6 m depth is colluvium deposits, consisting of unconsolidated sediments with thickness of 1–3 m and underlain layer of sandy to silty clay of 3 m in thickness. The layer of top soil is highly permeable, leading to easy infiltration to below silty clay layer, which is low permeability. This can make the subsurface susceptible to flow or slide on steep slopes. Under the colluviums lie a moderately weathered/fractured sandstone consisting of sand and gravel layer. This layer has approximately a thickness of 3 m and lies over a clay with weathered sandstone. Furthermore, the groundwater table is present in this layer. Under this layer is sandstone bedrock at a depth of approximately 16 m.

**Table 1:** Lithological description of the borehole near the research area (blue circle in Fig. 2).

Explanations	Depth
**Layer 1**: Colluvium sediments - Unconsolidated materials: dry sand, gravel, silt and laterite - Sandy to silty clay	0–3 m 3–6 m
**Layer 2**: Moderately weathered bedrock - Consolidated materials (compact sand and gravel) - Grey clay and the groundwater can be observed	6–9 m 9–16 m
**Layer 3**: Bedrock - Sandstone	Under 16 m

m (meter).

**Fig. 3:**
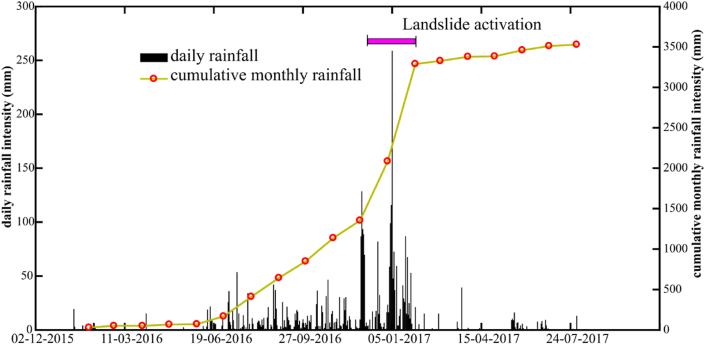
Daily and cumulative monthly rainfall intensity at Thungsong landslides from January 2016 to July 2017.

This research area is in a region of high rainfall intensity [39]. According to the Köppen system [40], the climate type of the study area is classified in Tropical Monsoon Climate with annual rainfall intensity of 1800–2200 mm. This climate type is susceptible to triggering landslides [41]. The raining season begins from the mid of May to mid-January. The highest rainfall intensity with daily rainfall over 200 mm is during October and November influenced by northeast monsoons [42]. According to landslide database in Thailand [43], there were the major landslide events in this area, which occurred in January 2017. During that time, the cumulative monthly rainfall intensity, acquired from Royal Irrigation Department of Thailand exceeded 3000 mm (Fig. 3) [44]. The landslides destroyed many assets, such as, highways, houses, and rubber plantations, which is an economic crop in southern Thailand [45]. In this study, a landslide (light green circle in Fig. 2) is investigated with the SRT and ERT methods. This landslide is triggered by extremely heavy rainfall, which infiltrates and increases pore water pressures and reduces the shear strength in the residual soil when soil suctions are decreased or dissipated [30,31,35,46].

**Fig. 4:**
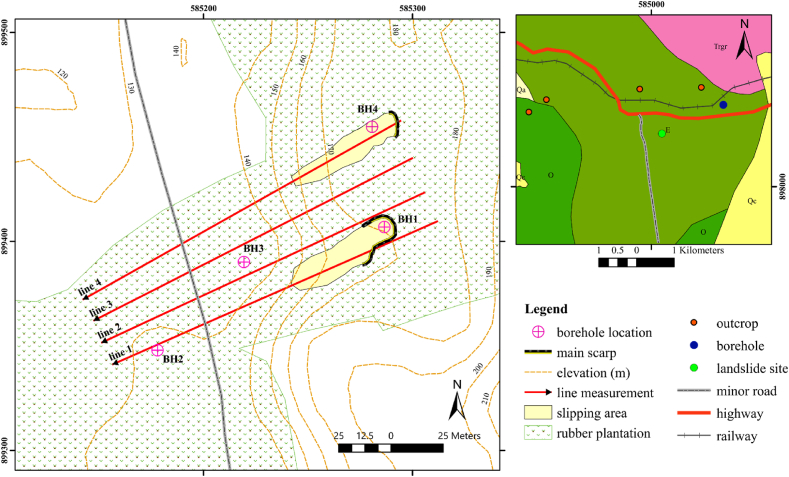
Map of the geophysical survey in the study area. Line 1 and line 4 cover the area of landslides revealing two main back scarps in January 2017 and are pseudo-parallel to line 2 and line 3.

The combination of climatic parameters leads to high chemical weathering, which ultimately gives rise to the formation of residual soil. The rainfall infiltration into the residual soil and its impact on slope instability has garnered significant attention in the past few years [47,48]. The landslide is located on the residual soils, which are the Quaternary colluvium deposits [49,50] underlain with sandstone bedrock. The colluvium deposits dislocated and slipped from a main back scarp (at the top of the slope) downward to toe of slope with distance of roughly 60 m. This landslide consists of two main scarps (Fig. 4). From the site survey, a width of the one main back scarp is 30 m and another width is 15 m. The depths of sliding surface are the same with approximately 2–4 m classified as shallow landslide. A few old fissures are discovered on upslope beyond the main scarps. In many areas of the sliding consist of the colluvium deposits, which consist of silty clay, displaced blocks of boulders gravel, lateritic soil, dry sand, and gravel. Moreover, many tensions cracks and a minor scarp are also found at further downhill next to the main scarp, which is covered by landslide deposits. The sliding deposits are covered by overgrown vegetation during raining season, which make the SRT and ERT surveys risky and laborious. There is a drainage canal at the base of the landslide, where water can discharge.

## 3 Geophysical data acquisition and data processing

P-wave SRT and ERT measurements were deployed in the research area along with line 1, line 2, line 3, and line 4 starting from northeast to southwest direction (Fig. 4). Lines 1 and 4 cover the region of landslides in January 2017 and exhibit a pseudo-parallel orientation to lines 2 and 3. The distance between each line is approximately 15 m. The sliding zones are situated at distances ranging from 23 m to 68 m along line 1 and between 3 m and 26 m along line 4 (Fig. 4). Moreover, there are four boreholes along with geophysical measurements (Fig. 4). The core loggings of drilled samples from the boreholes were used to support and validate geophysical data.

### 3.1 The SRT measurement and data processing

**Fig. 5:**
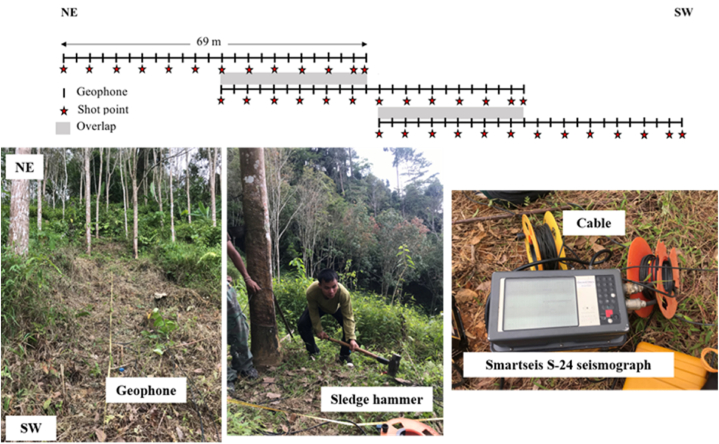
P-wave SRT layout (top) and SRT data acquisition at the research area (below).

The P-wave SRT was employed with four profiles with a Smartseis S-24 seismograph with 24 channels for data recording (Fig. 5 below). A 10 kg sledge hammer was used as the energy source. The twenty-four 14 Hz-geophones deployed from top to base of slope in NE-SW direction with 3 m spacing and the shot interval is 6 m (Fig. 5 top). Each shot point was stacked 5 times to enhance the signal-to-noise ratio. Each profile has the length of 141 m, which consists of 3 spreads with 2 overlaps of 33 m and each spread expands over 69 m (Fig. 5 top). The recording system was placed at the middle of the layout of the first spread. Every shot point was recorded a time length of 512 ms at the sampling rate of 0.25 ms.

**Fig. 6:**
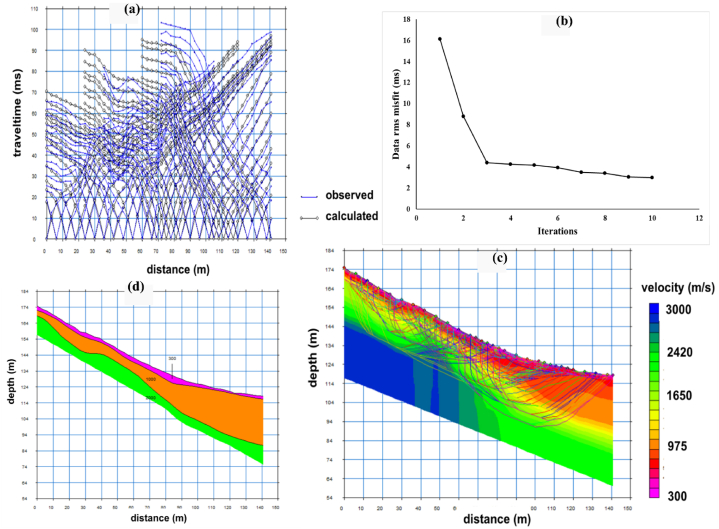
An example of the P-wave SRT inversion of line 4: (a) observed (blue) and calculated (black) traveltime curves for each shot gathers; (b) the curve between RMS and model iteration; (c) the final 2D depth-velocity model, with ray coverage superimposed; (d) the final layered model derived from the tomographic inversion. (For interpretation of the references to color in this figure legend, the reader is referred to the Web version of this article.)

The SRT data processing is based on SeisImager2D software package [51] consists of two steps, which are first break picking and inversion. The noise signals originating from traffic and wind were removed through frequency filtering before picking first arrival time. Additionally, a gain control was implemented to amplify and improve the seismic data quality. Following this, the first arrival times of recorded P-waves were identified for each shot point, and time-distance (t-x) curves were generated using the Pickwin. These t-x curves were then inverted to create a velocity model through inversion using the Plotrefa program. The inversion algorithm is related to the Non-linear Least Squares method [51]. The inversion method begins with the creation of an initial velocity model by time-term inversion and set parameters based on the first arrival travel time curve. The initial model exhibits larger cell sizes at greater depths compared to those near the surface [52]. Ray tracing method are applied through the model and traveltimes to calculate traveltimes, which are used to compare to measured traveltimes in terms of an RMS error. The model is iterative until the minimum difference between the calculated and measured traveltimes. An example of the observed and calculated traveltime curves for line 4 is shown in Fig. 6a with the RMS error of 3.0 ms (Fig. 6b). The 2D depth-velocity model and the ray coverage are also shown in Fig. 6c. This SRT model is converted to a layer model, that is better represent the layer of lithology (Fig. 6d). Moreover, the tomographic result can be also exported into an XYZ text file and reveals in 2D model using the Surfer software package version 14.0, which reveals in section 4 (SRT result).

### 3.2 The ERT measurement and data processing

**Fig. 7:**
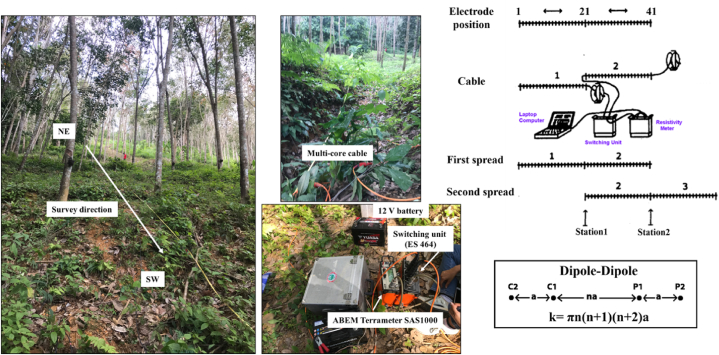
ERT data acquisition at the study area. The direction of field survey and the setup of the equipment are also shown in the photographs.

The 2D ERT surveys were performed using the ABEM Lund imaging system (Fig. 7). Each profile has the length of 180 m. The ERT data was collected using 61 electrodes with spacing of 3 m and connected to the ABEM Terrameter SAS1000 by multi-core cables. A dipole-dipole array (low right of Fig. 7) was selected throughout the surveys with dipole lengths (a) of 3, 6, and 9 and dipole separation (na) of 1a to 6a. There are many advantages of the dipole-dipole array: it is good in mapping horizontal changes [53] in landslide area; it reduces the impact of shallow geological variations, thereby improving the accuracy of deeper subsurface interpretations. An ES 464 (electronic switching unit) is connected to the ABEM SAS1000 for the selection of four electrodes in each measurement (Fig. 7). In the first spread, the ABEM SAS1000 and the ES 464 were positioned at the middle of the layout (station 1 in Fig. 7 left). Subsequently, the two cables were connected to the ES 464 and output to the connect the ABEM Terrameter SAS1000 (Fig. 7). After the measurement in the first spread finished, the first cable was displaced to connect at the end of the first spread known as the roll-along method to expand horizontally the survey area (the second spread in Fig. 7 left). Subsequently, the ABEM SAS1000 and the ES 464 were displaced to the center of the second spread and continued to measure (station 2 of Fig. 7 bottom left).

**Fig. 8:**
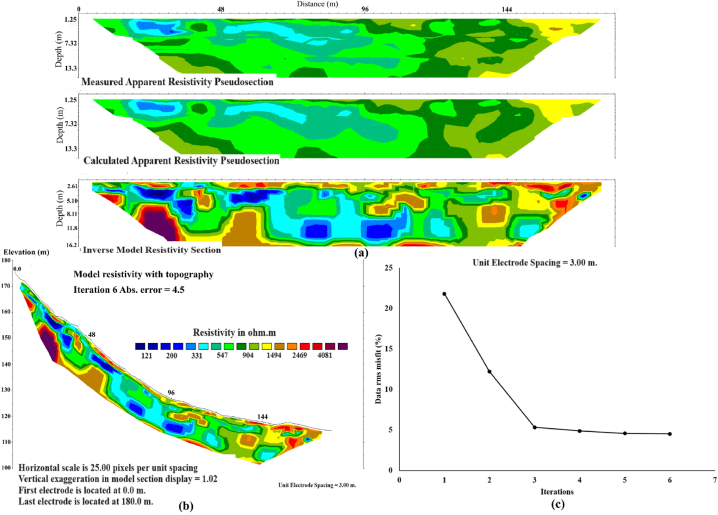
An example of the ERT inversion of line 4: (a) inversion result of line 4 produced by Res2Dinv software; (b) model resistivity for line 4 displayed with the true topography; (c) the curve between RMS and model iteration from ERT inversion.

Data processing is carried out by RES2DINV (Version 3.59), developed by Geotomo Software. The processing steps adhere to the methodology outlined by Loke and Barker (1996) [51], aiming to generate a 2D true resistivity model through an inversion process. Before inversion, the undesirable datum points were deleted. These points were recognized as outliers owing to poor electrode contact with the ground surface, often attributed to very dry soil and rock conditions. The topographic data obtained from field measurements was incorporated into the processing. During inversion, the apparent resistivity data was converted into a true resistivity model, providing insights into the subsurface’s depth. The inversion algorithm used in the software is based on the smoothness-constrained least-squares method [54,55] which can be expressed as,

(1) (J^T^J + u F)d = J^T^g,

where F = f_x_f_x_^T^ + f_z_f_z_^T^, f_x_ represents the horizontal flatness filter, f_z_ is the vertical flatness filter, J is the Jacobian matrix of partial derivatives, u is the damping factor, regulating the emphasis on model smoothness, d is the model perturbation vector denoting the alterations in model resistivity values, and g is the discrepancy vector encompassing the disparities between the calculated and measured apparent resistivity values. The magnitude of this vector is commonly expressed as the Root-Mean-Squared (RMS) value, which can be calculated as [56],

(2) $$\text{RMS}=\sqrt{\frac{1}{n^{2}}\sum_{i=1}^{n}{\left[\frac{\log\left(\rho_{{\text{meas}}_{i}}\right)‐\log\left(\rho_{{\text{cal}}_{i}}\right)}{\log\left(\rho_{{\text{cal}}_{i}}\right)}\right]}^{2},}$$

where $\rho_{\text{meas}}$ is measured apparent resistivity values, $\rho_{\text{cal}}$ is calculated apparent resistivity values, n is total number of measurement points, and i is measurement point. The inversion aims to reduce the RMS as it attempts to find a better model with each iteration [56]. However, Equation (1) constrains the change in the model resistivity values, d, to be smooth, but it does not guarantee to capture the shape of the boundaries between different geological features, capturing change in the resistivity values across boundaries in a realistic manner. Therefore, Equation (1) can be modified using a L_1_-norm smoothness-constrained optimization method, or a blocky inversion method. The L_1_ norm method exhibits lower sensitivity to noisy dataset. Further details of the L_1_ norm method are described by Loke et al. (2003) [57]. An example of the ERT inversion with a curve between RMS and model smoothness of line 4 is presented in Fig. 8. It contains three sections (Fig. 8a): the top section is the measured data plotted as a pseudosection, the middle section is the calculated data, and the bottom section shows the inversion model. The inversion model is displayed with topography in the final model section from the RES2DINV (Fig. 8b). This model can be exported into a XYZ format to plot as a 2D image using the Surfer software package version 14.0 which exhibits in section 4 (ERT result).

### 3.3 2D cross-plot analysis model

Cross-plot analysis has been widely utilized to evaluate and delineate geological formation and lithology in the oil and gas industry [[57], [58], [59]]. Many geophysical parameters from borehole logging, such as seismic velocity, nuclear radiation, and resistivity have been input to determine and characterize lithological reservoir [60]. Hayashi and Konishi (2010) have shown a cross-plot analysis of S-wave velocity and resistivity values for levee assessment [61]. The cross-plot analysis reveals the effectiveness on the evaluation of seepage and erosion of levee. Moreover, Wodajo et., al (2019) used cross-plot analysis of P-wave velocity and electrical resistivity values to evaluate vulnerability of a dam or levee [60]. The cross-plot analysis based on their values can classify the soil type and assess the degree of compaction of the dam or levee [60].

**Fig. 9:**
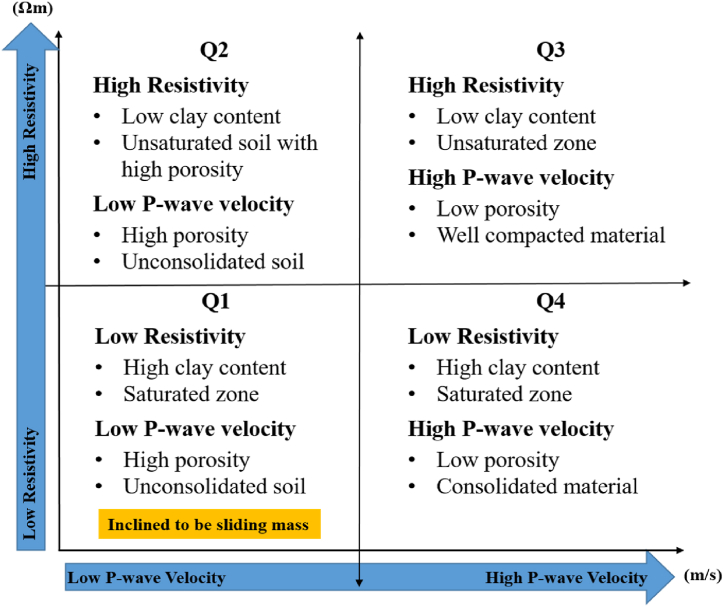
Schematic cross-plot graph of P-wave seismic velocity and electrical resistivity values with soil conditions in landslide area.

Here, a novel approach to cross-plot analysis based on the previous Hayashi model [60] has been introduced to enhance the resolution of subsurface structure in landslide area by integrating two distinct P-wave velocity and resistivity values. It involves examining the resistivity and P-wave velocity values extracted from the ERT and SRT models at identical points along all surveyed lines. These values are then plotted against each other on a cross-plot graph, where the x-axis represents P-wave velocity and the y-axis represents resistivity. The parameters are divided into four quadrants that relates to the relation between P-wave velocity and resistivity value attributing to landslide in this area (Fig. 9). Quadrant 1 (Q1) signifies low P-wave velocity and low resistivity, while Quadrant 2 (Q2) indicates low P-wave velocity and high resistivity. Quadrant 3 (Q3) points to high P-wave velocity and high resistivity, and Quadrant 4 (Q4) reflects high P-wave velocity and low resistivity.

There are three steps to produce the 2D cross-plot model. First, the SRT and ERT models for every profile are projected in the same axis of x (distance) and z (elevation). The resistivity and P-wave velocity values are picked at the same points on the ERT and SRT models. Any datum points that fall outside at the same x and z positions are removed from the data set. Subsequently, the graphical and datum point analyses are conducted to create a P-wave velocity-resistivity graph. During this stage, data points with values significantly larger than the average range are eliminated. The clustering process is then employed to sort the data based on the resistivity and velocity values that are interpreted with borehole data to define each quadrant in the velocity-resistivity graph. The threshold values for clustering data are chosen based on Sujitapan, 2023 [62]. The four quadrants (Q1-Q4) are allocated and arranged systematically, as shown in Fig. 9. Finally, all the data for each quadrant are exported to be plotted as a 2-D model. The resulting model is plotted according to the x and z positions of the data points.

## 4 The SRT and ERT results and interpretation

**Fig. 10:**
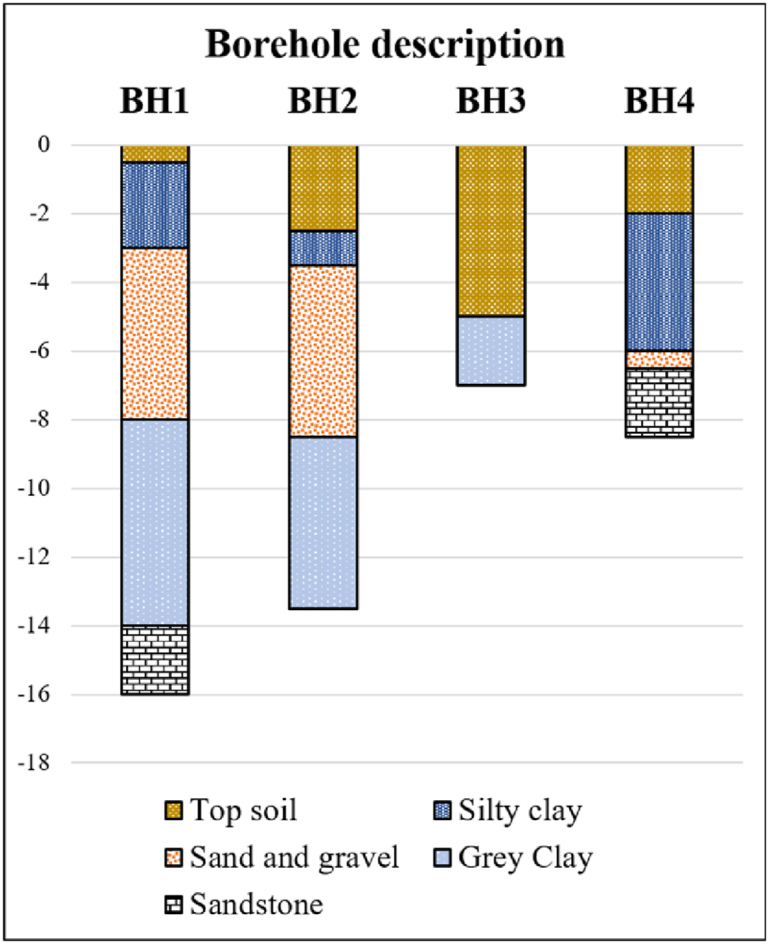
Borehole investigations (BH1–BH4) in this research area (the locations of boreholes is in Fig. 4).

After the data processing is completed, the ERT and P-wave SRT models are superimposed and compared with the borehole data from DGR (Table 1) and BH1–BH4 (Fig. 10) in this research area. The interpretations of the data are conducted by considering the lithology observed in the borehole and referring to published resistivity and seismic velocity values for different types of earth materials. Finally, the integration of two datasets in terms of 2D cross-plot model is then produced to analyze the interplay between the ERT and SRT data.

**Fig. 11:**
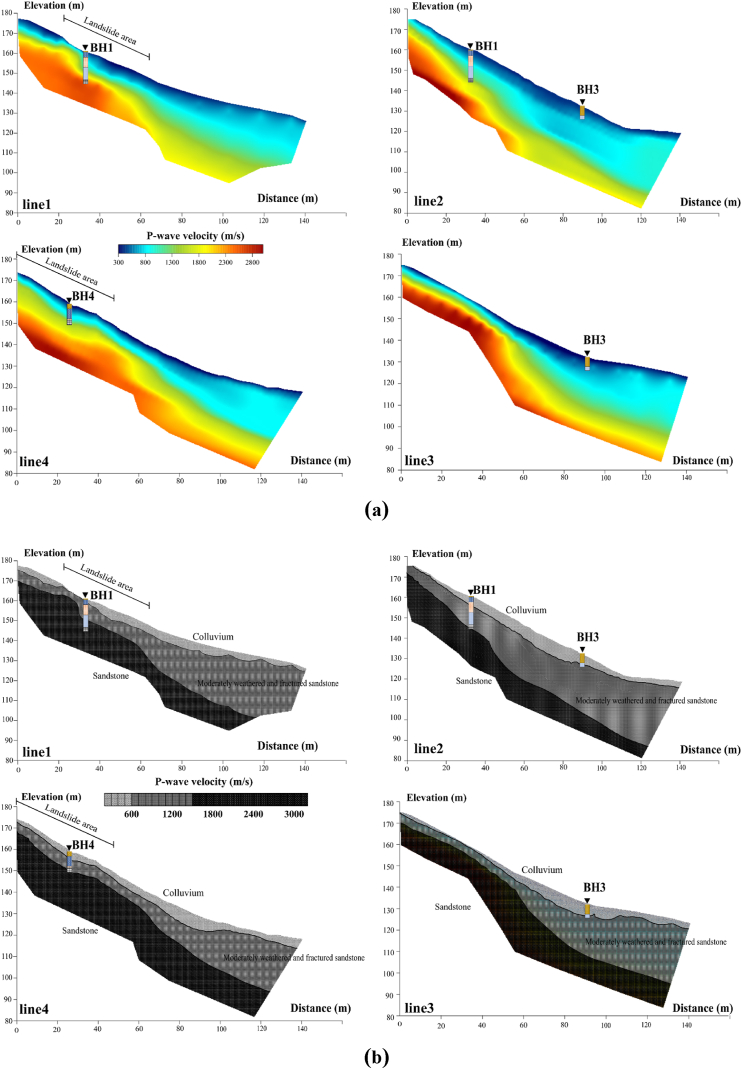
SRT results correlated with borehole data in this landslide area: (a) SRT inversion models; (b) SRT layered models.

The SRT inversion generated four SRT models, as depicted in Fig. 11a, with absolute RMS errors of 3.1, 2.3, 2.2, and 3.0 ms for lines 1, 2, 3, and 4, respectively. These tomographic models can be converted into four P-wave velocity layer models based on lithological data, as depicted in Fig. 11b. These layered models exhibit the presence of three distinct layers. The uppermost layer displays P-wave velocity ranging from 300 m/s to 600 m/s. This low P-wave velocity is indicative of the colluvium deposits. The depth of this layer varies from 0 m to 5 m. This layer constitutes the primary sliding material, aligning with the failure at the main scarp and exhibiting a steep angle at the top of the slope. The major sliding materials displaced roughly 50 m downslope from the visible scarp (crown) to the base of the slope in line 1, and about 30 m in line 4. Beneath the colluvium layer is interpreted as moderately weathered sandstone with P-wave velocities in the range of 600 m/s to 1500 m/s, correlated to the BH1 and BH2 in line 1. The bottom layer, interpreted as sandstone bedrock displays P-wave velocities greater than 1500 m/s. This layer is correlated with the BH1 and BH4 in lines 1 and 4. The moderately weathered sandstone and the sandstone layer adhere to the surface nearby the top of the slope. In the landslide regions (lines 1 and 4), the sandstone bedrock exhibits a notably steep slope angle at the back scarp, potentially triggering landslide event. The rupture surfaces in lines 1 and 4 can be observed at the depth of roughly 2–3 m.

**Fig. 12:**
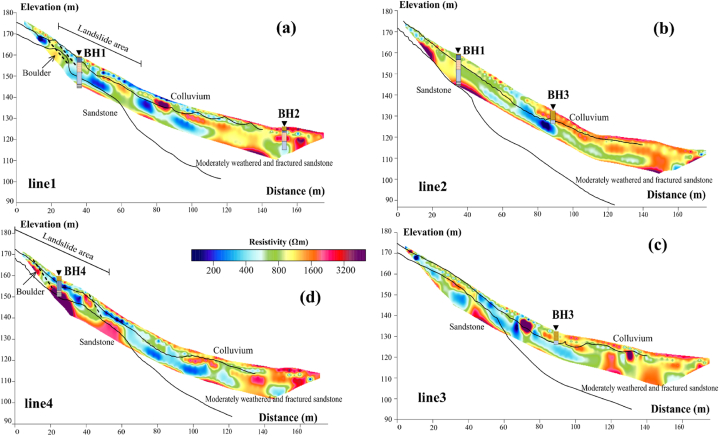
ERT inversion models correlated with borehole in this landslide area: (a) line 1; (b) line 2; (c) line 3; (d) line 4. The black line represents the layer boundary based on SRT results. The black dash line represents the fracture zones.

The ERT models are displayed with the length of 180 m (Fig. 12). The RMS errors for lines 1, 2, 3, and 4 are 6.6%, 7.7%, 7.2%, and 4.5%, respectively. Each model is separated into three layers according to the SRT layer models (Fig. 12). The colluvium deposits (the top layer) exhibits relatively low resistivity values ranging from 60 to 250 Ωm, especially at the top of the slope in lines 1 and 4 (the landslide regions). The relatively low resistivity is indicative of moist sandy to silty clay (low porosity material), which is correlated to the BH1 and BH4. Conversely, relatively high resistivity values ranging from 600 to 2500 Ωm are indicative of dry sand and highly weathered sandstone fragments (high porosity material), correlated to the BH2 and BH3 at the toe of slope in lines 1–4. The presence of clay zones aligns consistently with the landslide material, corresponding to the sliding position on the slope in lines 1 and 4 (Fig. 12a and d). The layer below is indicative of the moderately weathered sandstone layer. The relatively low resistivity (less than 250 Ωm) regions at the middle and toe of the slope are correlated to silty clay and grey clay with the saturation layer in the BH1–BH3. On the contrary, the relatively high resistivity regions (greater than 600 Ωm) are correlated to sand and gravel layer (low water and clay content) in the BH1, BH3, and BH4. This layer is adjacent to the bottom layer, which demonstrates very high resistivity values, exceeding 1500 Ωm. The bottom layer is correlated to sandstone bedrock layer in the BH1 and BH4, which is possibly found in the top of slope.

## 5 Discussion

The integration of the 2D ERT and P-wave SRT results offers valuable complementary information for evaluating the structure of the landslide at the study site. In this assessment, a thorough comparison and calibration are conducted between the geological structures and geophysical data (including P-wave velocity and resistivity values) with the existing borehole data. This calibration process enables a comprehensive analysis that takes into account both the geophysical measurements and the geological features observed in the boreholes.

The geological structures in this landslide area can be divided into three layers obtained from the SRT layered models and boreholes. The subsurface characteristics of the study area are revealed through the analysis of 2-D resistivity and seismic profiles. Within this area, a failure zone has been identified and characterized by the presence of a fracture zone (black dash lines in lines 1 and 4 of Fig. 12a and d) containing boulders in lines 1 and 4, which has the potential to trigger mass movements. The occurrence of mass movement can be attributed to a region of low resistivity, indicating high saturation. This highly saturated zone is primarily composed of sandy and sandy silt soil in colluvium, which further contributes to the slope failure. The uninterrupted infiltration of surface water, combined with heavy rainfall, seeps into the fracture area and accumulates, exacerbating the slope instability due to the high permeability and porosity of the soil. The disturbance of soil properties, including variations in porosity and permeability, increases the effective stress within the soil, ultimately triggering mass movements in the unstable slope. To develop the visualization of features in slipping zone, an in-depth examination and comparison of geological features, resistivity values, and velocity values are conducted in the next section.

### 5.1 2D cross-plot analysis of P-wave velocity and resistivity value

**Fig. 13:**
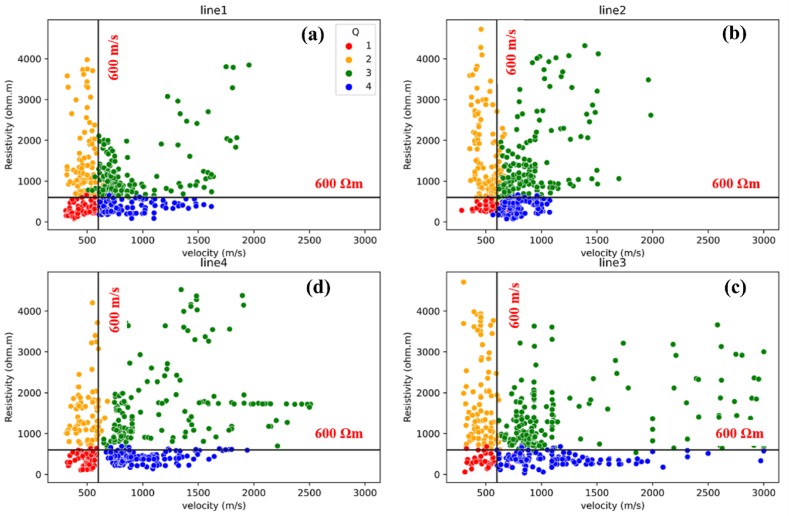
2D cross-plot graphs of P-wave velocity and resistivity values in this landslide area: (a) line 1; (b) line 2; (c) line 3; (d) line 4. The black line is the boundaries of threshold values for each quadrant.

In this section, the interaction between two geophysical datasets (resistivity and P-wave velocity values) and geological features is thoroughly investigated. From the results of SRT and ERT, the landslide slip surfaces are triggered by saturated zones the colluvium layer with the depth of 5 m. Consequently, the values of P-wave velocity and resistivity in the study area to separate data points into four quadrants are the P-wave velocity of less than 600 m/s and resistivity of less than 600 Ωm (Fig. 13). This specific value acts as a threshold for determining the likelihood of landslide events. The four quadrants indicating four properties of the subsurface are Q1 (≤600 Ωm, ≤600 m/s), Q2 (≥600 Ωm, ≤600 m/s), Q3 (≥600 Ωm, ≥600 m/s), and Q4 (≤600 Ωm, ≥600 m/s). The Q1 delineates an area marked by extensive weathering, leading to diminished resistivity and velocity values. This sector indicates a potential susceptibility to mass movements or slope failure. The Q2 exhibits reduced velocity and elevated resistivity values, implying the existence of extensively weathered materials along this profile. The Q3 signifies the presence of compact materials or boulders. The dominant region in the plot is the Q4, indicating a higher clay content and greater water content in moderately weathered or fractured sandstone.

**Fig. 14:**
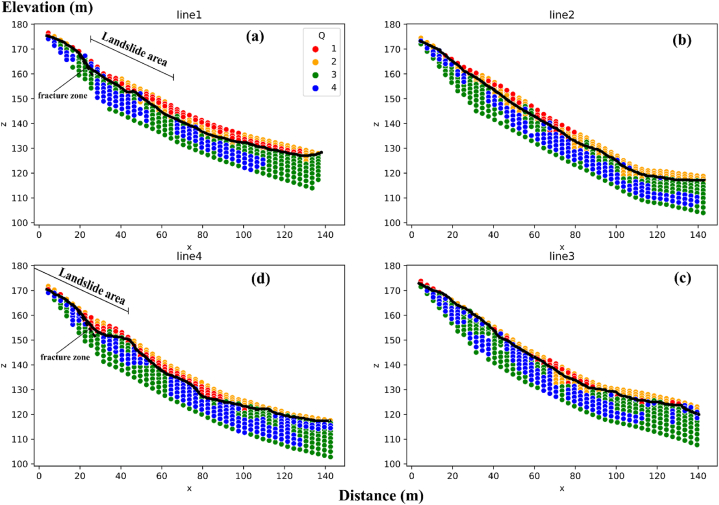
2D cross-plot models of P-wave velocity and resistivity values in this landslide area: (a) line 1; (b) line 2; (c) line 3; (d) line 4. The black line is the suspected sliding plane. The black dash line is fracture zones.

As the results of the 2D cross-plot graph (Fig. 13), a 2D cross-plot model (Fig. 14) is produced by extract values based on their relative depths and positions for each profile. This is the improvement in cross-plot analysis to visually enhance and represent the comprehensive structure of subsurface. Within this model, a sliding plane (black line in Fig. 14) is identified at a depth of less than 5 m, primarily dominating the first and second quadrants (Q1 and Q2). The 2D cross-plot models show that upper sliding plane layer exhibits the colluvium layer. Within the colluvium layer, two distinct groups of resistivity values are observed. The first group displays relatively high resistivity (more than 600 Ωm), classified to Q2 (represented by orange circles in Fig. 14). These high resistivity values indicate the presence of very dry and poorly consolidated materials near the surface. The second group exhibits relatively low resistivity values (less than 600 Ωm), classified to Q1 (represented by red circles in Fig. 13). These lower resistivity values signify the presence of silty clay material, which is consistent with observations from the boreholes. It is important to note that the colluvium layer, primarily composed of these materials, is highly susceptible to landslides, particularly in areas where relatively high clay contents are found (such as line 1 and line 4).

Under the sliding plane is identified as sandstone that is moderately to less weathered or fractured, exhibiting a higher P-wave velocity greater than 600 m/s. Within this layer, two distinct groups of resistivity values can be observed in Q3 and Q4. The first group (Q3) displays comparatively high resistivity values greater than 600 Ωm (represented by green circles in Fig. 14), suggesting the presence of sand and gravel and a compact sandstone layer. The second group (Q4) exhibits relatively low resistivity values less than 600 Ωm (represented by blue circles in Fig. 14), indicating a higher clay content and greater water content. Typically, the sand and gravel component is located in the upper part of this layer, while the clay-rich and water-saturated layer is interpreted as the water table and is found in the lower part.

Furthermore, the 2D cross-plot models can be divided into three zones by distance. The first zone covers the uppermost part of slope, spanning a distance of 0–65 m. The slope in this zone is relatively steep, featuring slope angles of approximately 40°. This zone encompasses the active landslide regions found in lines 1 and 4. The occurrence of landslides in these areas can be attributed to the combination of comparatively steep slopes and a prominent concentration of silty clay content (Q1) within the landslide zone (lines 1 and 4 in Fig. 14a and d). The displaced material in these landslides consists of colluvium, with thicknesses varying from 2 to 5 m as shown in the cross-plot model. The slip surface, depicted by a black dashed line in Fig. 14a and d, can be observed in the back scarps of both line 1 and line 4. These landslides took place in January 2017, coinciding with heavy rainfall. The cumulative monthly rainfall during that period exceeded 3000 mm, as shown in Fig. 3. The second zone covers the middle part of slope, spanning a distance of 65–127 m. In this zone, there is a lot of moderately to less weathered/fractured sandstone (Q3), which overlays a deeper layer of sandstone bedrock. Above the sandstone bedrock, there is a layer of grey clay (Q4), which contains groundwater flowing from the top of the slope and permeates downward through the overlying layers. The last zone is in the base of the slope spanning a distance of 127–170 m. The uppermost layer in this zone is the Q2, which is distinguished by comparatively high resistivities (over 600 Ωm) but comparatively low seismic velocities (less than 600 m/s). This layer comprises very dry, poorly consolidated sediments with minimal signs of silty clays. Below the colluvium layer, there is sand and gravel in moderately weathered/fractured sandstone layer. In contrast to the second zone, there is inadequate evidence of deeper clays and the presence of water table within this zone. The sandstone bedrock is situated at a depth that is not observable within this particular zone.

**Fig. 15:**
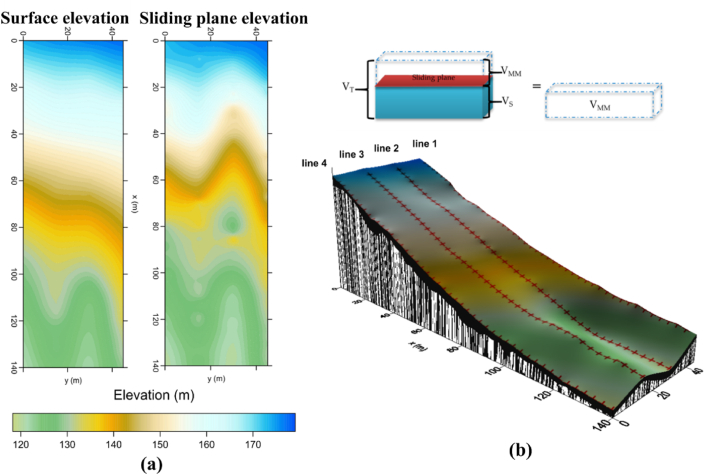
Volume calculation of sliding materials in this research area*: (*a*)* the elevation maps of the surface and sliding plane; *(*b*)* volumetric calculation of mass movement *(*solid black area*)* in this landslide area.

From the 2-D cross-plot model, the boundaries of suspected sliding plane (black color line in Fig. 14) can be employed to calculate the volume of sliding material (black color in Fig. 15b). The volume is calculated using digital elevation maps (Fig. 15a) created by interpolations methods [10]. The calculated volume of the mass sliding plane (V_MM_) is determined by the subtraction of the volume of entire area (V_T_) to the volume of under slipping plane (V_s_) (Fig. 15b top). In this study, the surface elevation map (Fig. 15a left) is used to calculate the total volume of study area (V_T_) and the elevation map of sliding plane (Fig. 15a right) digitized from 2D cross-plot model is used to determine the volume of under failure plane (V_s_). This calculated volume is based on the numerical methods, which are Trapezoidal Rule, Simson’s Rule, and Simpson’s 3/8 Rule [63,64] in the volume function of the Surfer11 software. The difference in the volume calculations by these three methods can measure the accuracy of the volume calculations. When the three volume calculations are close together, the true volume is reasonably close to these values. The true volume can be calculated from the average of the three values [65]. In this study, the average volume calculation from three methods is approximately 33447.76 m^3^ with standard variation of 0.17 m^3^, which reveals low uncertainty of this volume calculation. This value represents the volume of the sliding material in this landslide area (V_MM_). However, the choice of the location of the landslide sliding surface is definitely affected by uncertainty. Therefore, the reliable volume of sliding mass depends on the elevation map of failure plane.

In summary, this study is successful to assess the slope instability in the study region. The cross-plot analysis demonstrates an intuitive approach to integrate P-wave velocity and resistivity methods. These geophysical methods offer in-depth insights into landslide characteristics, and their findings are reinforced through validation with parameters of hydrogeology and geotechnique, bolstering the interpretation of geophysical results. The combination of cross-plot analysis and the development of a 2-D cross-plot model proves to be an informative approach to characterize and improve subsurface imaging. However, comparing to other works, such as Whiteley et al. (2021a) [66] and Marzan et al. (2021) [67] this work reveals the weakness of the methodological approaches in clustering process and the uncertainty in position of sliding plane. Consequently, this study can be developed in clustering process using machine learning to reduce uncertainty in subsurface characterization of landslide. For example, the ERT and SRT results from this study can integrate with a Gaussian Mixture Model (GMM) [66], which is a type of unsupervised machine learning, to categorize the geophysical data into clusters based on shared measurement ranges and trends [65].

## 6 Conclusions

By a combination of the SRT and ERT techniques, an in-depth knowledge of the internal structure and deformation mechanisms of the Thungsong landslides has been achieved. The result reveals that weathering and distress have led to the weakening of soil profiles. This is characterized by velocities below 600 m/s and resistivity values below 600 Ωm, which serve as the threshold for failure. A combination of multiple factors contributes to the occurrence of landslides in the Thungsong area. The steep slopes in the region render the overlying colluvium unstable. The presence of abundant clay material near the top of the slope adds to the susceptibility, as clay possesses ductile characteristics and readily absorbs water. Additionally, a weakness plane exists at the base of the colluvium, comprising sand and gravel. The region’s vulnerability to heavy rainfall further exacerbates the situation, leading to seasonal landslides.

The identification of the sliding plane in 2D cross-plot model obtained from SRT and ERT results is crucial for assessing the failure zone across all profiles. The estimation volume of the sliding mass can be derived from this sliding plane. The integrated approach improves the interpretation of geophysical results and contributes to a better understanding of the subsurface conditions. Furthermore, the 2-D cross-plot model can develop to produce a landslide ground model, which provides general subsurface insights with minimal prior information, proving vital in disaster risk reduction situations and urgent assessments of unstable landslide formations and the ensuing chain of disasters, as highlighted by Whiteley et al. (2021b) [68]. It also serves as an essential source of preliminary data for the development of early warning systems tailored to slope-scale landslides.

## Funding

This research was funded by 10.13039/501100010034Walailak University (10.13039/501100010034WU), grant number “WU65254”

## Data availability statement

Data associated with this study will be made available on request.

## CRediT authorship contribution statement

**C. Sujitapan:** Writing - review & editing, Writing - original draft, Methodology, Investigation. **J.M. Kendall:** Data curation. **J.E. Chambers:** Validation, Conceptualization. **S. Yordkayhun:** Resources.

## Declaration of competing interest

The authors declare that they have no known competing financial interests or personal relationships that could have appeared to influence the work reported in this paper.
